# Draft Genome of Cladophialophora immunda, a Black Yeast and Efficient Degrader of Polyaromatic Hydrocarbons

**DOI:** 10.1128/genomeA.01283-14

**Published:** 2015-01-29

**Authors:** Katja Sterflinger, Ksenija Lopandic, Barbara Blasi, Caroline Poynter, Sybren de Hoog, Hakim Tafer

**Affiliations:** a VIBT-Extremophile Center, University of Natural Resources and Life Sciences, Vienna, Austria; b CBS Fungal Biodiversity Center, Utrecht, The Netherlands

## Abstract

The fungal genus Cladophialophora comprises many species which cause severe and even fatal infections in humans as well as environmental strains able to degrade polyaromatic hydrocarbons. The draft genome of Cladophialophora immunda presented here is the first whole-genome sequence within this important genus.

## GENOME ANNOUNCEMENT

Cladophialophora immunda is a representative of the black yeasts, a morphological group of ascomycetes characterized by melanin pigmentation and the ability to reproduce by branched chains of ellipsoidal to fusiform conidia. The genus Cladophialophora belongs to the ascomycetes and forms two phylogenetic clades (*Carrionii* and *Bantiana*) in the family of *Herpotrichiellaceae*, order *Chaetothyriales*. Together with the black yeast genus *Exophiala* and the teleomorph *Capronia*, species of Cladophialophora show a high prevalence in human infections, ranging from mild cutaneous infection to subcutaneous phaeohyphomycosis and to fatal encephalitis (1). The infection pathways and the virulence factors are not yet understood. Cladophialophora immunda is of special medical and biotechnological interest because it is frequently isolated both from humans and from contaminated soil and it was shown to grow on polyaromatic hydrocarbons (PAHs) as the sole carbon source (2). For this reason C. immunda is one of the most important candidates for bioremediation and for application in biofilters (3). The ability to degrade hydrocarbons and the pathogenic potential seem to be connected within the group of black yeasts. It was speculated that this is due to the chemical nature of aromatic rings both in PAH and in human neurotransmitters (4). Thus, the genome sequence of C. immunda is an important basis (i) for a deeper understanding of virulence and pathogenicity and (ii) for the understanding of possible biochemical pathways involved in biodegradation of PAHs.

Genomic DNA was isolated from Cladophialophora immunda (strain CBS 110551 from a gasoline station) grown on 2% malt extract agar using a cetyltrimethylammonium bromide (CTAB)-based protocol. Elimination of melanin from DNA was performed by two phenol-chloroform purification steps. Genome sequencing was carried out using the ION Proton Technology (Ion AmpliSeq Library preparation kit, Template OT2 200 kit, Ion PI Sequencing 200 kit, and Ion PI chip kit V2; Life Technologies, Carlsbad) following the instructions of the producers and achieving a total of 13.7 G with a median read length of 164 bp resulting in ~314× average coverage. The assembly consists of 466 contigs, with an average size of 89,238 bp, an *N*_50_ of 241,757 bp, and a total size of 41,585,216 bp. The presence of a putative genome duplication was investigated. For each of the 246 single-copy genes annotated in Saccharomyces cerevisae (5), the corresponding protein sequence was blasted against the genome with tblastn (6). The second lowest *P* value was saved, and the resulting *P*-value distribution was compared to the corresponding *P-*value distribution in *Hortaea werneckii*, a fungus that has undergone a recent genome duplication (7), as well as the *P*-value distribution in Exophiala dermatitidis. The similarity of the three distributions was assessed with the Wilcoxon test, indicating that the *P*-value distribution of C. immunda is much closer to that of E. dermatitidis (*P* = 0.23) than to that of H. werneckii (*P* <10^−16^). This indicates that the genome of C. immunda did not undergo genome duplication.

### Nucleotide sequence accession numbers.

The whole-genome shotgun project has been deposited at DDBJ/EMBL/GenBank under the accession number JSEJ00000000. The version described in this paper is version JSEJ01000000.
